# Supplementary material for: Dimethylformamide Inhibits Fungal Growth and Aflatoxin B1 Biosynthesis in Aspergillus flavus by Down-Regulating Glucose Metabolism and Amino Acid Biosynthesis
Source: Toxins (Basel). 2020 Oct 29;12(11):683. doi: 10.3390/toxins12110683 (PMC7692752; doi:10.3390/toxins12110683)
Supplement: Supplementary file 1 [file toxins-12-00683-s001.pdf]

Supplementary Materials: Dimethylformamide Inhibits Fungal Growth and Aflatoxin B1 Biosynthesis in *Aspergillus flavus* by Down-regulating Glucose Metabolism and Amino Acid Biosynthesis

Lin Pan, Peng Chang, Jing Jin, Qingli Yang and Fuguo Xing

Table S1. Transcriptional activity of genes involved in *A. flavus* carbon metabolism.

Gene ID	CK* (FPKM)	D1* (FPKM)	Log_2 D1/CK	Annotated Gene Function
AFLA_101470	1.44	0.040	−5.15	glyceraldehyde-3-phosphate dehydrogenase, putative
AFLA_041570	387.62	28.23	−3.78	conserved hypothetical protein
AFLA_052400	316.70	104.23	−1.60	isocitrate lyase AcuD
AFLA_028130	15.99	5.97	−1.42	thermoresistant gluconokinase family protein
AFLA_086400	402.88	178.47	−1.17	isocitrate dehydrogenase Idp1, putative
AFLA_085400	1255.25	582.91	−1.11	phosphoglycerate mutase, 2, 3-bisphosphoglycerate-independent
AFLA_069370	3560.94	1676.10	−1.09	phosphoglycerate kinase PgkA, putative
AFLA_119290	676.31	323.46	−1.06	Phosphofructokinase, putative
AFLA_047080	164.12	85.29	−0.94	cytosolic hydroxymethyltransferase, putative
AFLA_026470	263.66	141.33	−0.89	aspartate aminotransferase, putative
AFLA_041840	98.10	54.49	−0.85	glycine dehydrogenase
AFLA_046740	150.91	85.02	−0.83	NADP-dependent malic enzyme MaeA
AFLA_105320	166.68	98.79	−0.75	phosphoserine aminotransferase
AFLA_037910	678.05	414.33	−0.71	NAD^+-isocitrate dehydrogenase subunit I
AFLA_027310	20.02	12.37	−0.69	fructose-1, 6-bisphosphatase Fbp1, putative
AFLA_073260	37.37	23.13	−0.69	Hexokinase, putative
AFLA_025100	9433.07	5907.44	−0.68	glyceraldehyde 3-phosphate dehydrogenase GpdA
AFLA_068840	48.32	30.87	−0.65	glycine cleavage system T protein
AFLA_091270	210.94	139.70	−0.59	fumarate hydratase, putative
AFLA_111650	78.81	52.66	−0.58	threonine dehydratase, biosynthetic
AFLA_085420	537.59	361.44	−0.57	serine hydroxymethyl transferase, putative
AFLA_049390	210.48	141.58	−0.57	malate synthase AcuE
AFLA_134130	47.00	31.73	−0.57	methylenetetrahydrofolate reductase, putative
AFLA_054750	738.72	501.26	−0.56	malate dehydrogenase, NAD-dependent
AFLA_007020	885.66	616.47	−0.52	citrate synthase (Cit1), putative
AFLA_067450	195.06	136.01	−0.52	glycine cleavage system H protein
AFLA_021700	99.45	69.65	−0.51	Aminotransferase, class V, putative
AFLA_100700	115.97	83.77	−0.47	phosphoserine phosphatase
AFLA_044820	488.47	354.15	−0.46	glucose-6-phosphate isomerase
AFLA_048020	188.81	137.94	−0.45	D-3-phosphoglycerate dehydrogenase
AFLA_034050	1139.95	847.92	−0.43	mitochondrial aconitate hydratase, putative
AFLA_048610	274.66	207.49	−0.40	succinyl-CoA synthetase alpha subunit, putative
AFLA_115170	385.52	291.89	−0.40	succinyl-CoA synthetase beta subunit, putative
AFLA_018850	390.74	302.65	−0.37	isocitrate dehydrogenase, NAD-dependent
AFLA_072370	0.08	0.06	−0.35	Hexokinase, putative

AFLA_083880	417.48	339.18	−0.30	succinate dehydrogenase subunit CybS, putative
AFLA_045020	150.27	122.81	−0.29	aspartate transaminase, putative
AFLA_090000	0.49	0.43	−0.20	conserved hypothetical protein
AFLA_059570	0.25	0.22	−0.16	conserved hypothetical protein

* CK=Control; D1=1% dimethylformamide.

Table S2. Transcriptional activity of genes involved in *A. flavus* MAPK pathway, Oxylipins, GPCRs and OSR.

Gene ID	gene	CK* (FPKM)	D1* (FPKM)	Log_2 D1/CK	Annotated Gene Function
AFLA_062500	*maf1*	80.24	71.00	−0.17	mitogen-activated protein kinase MAF1
AFLA_083380	*pbs2*	49.22	44.29	−0.15	MAP kinase kinase (Pbs2), putative
AFLA_048880	*ste11*	16.98	15.66	−0.11	MAP kinase kinase kinase Ste11
AFLA_103480	*ste7*	21.33	20.04	−0.09	MAP kinase kinase Ste7
AFLA_034170	*fus3*	116.05	155.35	0.42	MAP kinase FUS3/KSS1
AFLA_035530	*ste20*	42.19	50.59	0.26	serine/threonine kinase Ste20
AFLA_073630	*ste20-like*	18.95	21.86	0.20	serine/threonine protein kinase, Ste20-like
AFLA_021030		24.99	22.37	−0.15	serine/threonine protein kinase, putative
AFLA_052570	*mpkA*	67.01	64.48	−0.05	MAP kinase MpkA
AFLA_051240	*mkk2*	99.25	77.87	−0.35	MAP kinase kinase (Mkk2), putative
AFLA_031560	*bck1*	17.71	18.20	0.03	MAP kinase kinase kinase (Bck1), putative
AFLA_100250	*cat*	1.39	0.72	−0.93	catalase Cat
AFLA_090690	*cat1*	500.21	555.65	0.15	mycelial catalase Cat1
AFLA_122110	*cat2*	12.65	17.77	0.48	bifunctional catalase-peroxidase Cat2
AFLA_056170	*catA*	47.28	108.67	1.20	spore-specific catalase CatA
AFLA_099000	*sod1*	311.11	209.12	−0.57	Cu, Zn superoxide dismutase SOD1
AFLA_033420	*mnSOD*	1844.59	2033.04	0.14	Mn superoxide dismutase MnSOD
AFLA_031340	*atfA*	68.74	52.93	−0.37	bZIP transcription factor (AtfA), putative
AFLA_094010	*atfB*	94.74	98.13	0.05	bZIP transcription factor (Atf21), putative
AFLA_129340	*ap-1*	172.52	103.13	−0.74	bZIP transcription factor AP-1
AFLA_110650	*msnA*	147.14	152.03	0.04	C_2H_2 transcription factor (Seb1)
AFLA_091490	*mtfA*	42.87	81.01	0.91	C_2H_2 finger domain protein
AFLA_030580	*pacC*	138.05	116.60	−0.24	C_2H_2 transcription factor PacC
AFLA_034540	*srrA*	52.96	65.05	0.29	stress response transcription factor SrrA/Skn7, putative
AFLA_062210	*sskA*	42.65	41.37	−0.04	response regulator, putative
AFLA_068590	*sskB*	22.86	28.84	0.33	MAP kinase kinase kinase SskB, putative
AFLA_099500	*sakA1*	8.46	13.53	0.67	MAP kinase SakA
AFLA_061090	*sakA2*	11.34	40.50	1.83	MAP kinase SakA
AFLA_026790	*ppoA*	23.61	58.07	1.29	fatty acid oxygenase PpoA, putative
AFLA_120760	*ppoB*	0.00	0.00	Up	fatty acid oxygenase, putative
AFLA_030430	*ppoC*	1.74	4.36	1.32	fatty acid oxygenase PpoC, putative
AFLA_025100	*gpdA*	9433.07	5907.44	−0.67	glyceraldehyde 3-phosphate dehydrogenase GpdA
AFLA_046760	*gfdB*	29.62	10.94	−1.43	glycerol 3-phosphate dehydrogenase (GfdB), putative
AFLA_060740	*gprA*	6.13	5.61	−0.12	mating-type alpha-pheromone receptor PreB
AFLA_061620	*gprB*	9.84	11.96	0.28	a-pheromone receptor PreA

AFLA_074150	*gprC*	20.10	13.97	−0.52	conserved hypothetical protein
AFLA_135680	*gprD*	15.89	9.93	−0.67	G protein-coupled receptor GprD
AFLA_006880	*gprF*	29.26	33.18	0.18	PQ loop repeat protein
AFLA_067770	*gprG*	58.95	28.22	−1.06	PQ loop repeat protein
AFLA_006920	*gprH*	1.54	3.47	1.16	cAMP receptor-like protein
AFLA_127870	*gprJ*	37.85	32.38	−0.22	vacuolar membrane PQ loop repeat protein
AFLA_009790	*gprK*	0.08	0.40	2.26	conserved hypothetical protein
AFLA_075000	*gprM*	11.82	33.67	1.51	conserved hypothetical protein
AFLA_032130	*gprO*	40.56	42.44	0.06	hemolysin-III channel protein Izh2, putative
AFLA_088190	*gprP*	45.71	44.28	−0.04	IZH family channel protein (Izh3), putative
AFLA_023070	*gprR*	100.49	80.17	−0.32	integral membrane protein
AFLA_006320	*gprS*	40.64	34.30	−0.24	PQ loop repeat protein
AFLA_117970	*nopA*	1380.92	1261.75	−0.13	opsin, putative
AFLA_039130	*sln1*	189.95	235.23	0.30	sensor histidine kinase/response regulator TcsB/Sln1, putative
AFLA_045720	*mak10*	22.95	17.07	−0.42	amino-acid N-acetyltransferase subunit Mak10, putative
AFLA_112280	*mak11*	40.78	46.82	0.19	60S ribosome biogenesis protein Mak11, putative
AFLA_018940	*mak32*	13.76	16.96	0.30	PfkB family carbohydrate kinase (Mak32), putative
AFLA_106830	*tcsA*	16.58	19.07	0.20	sensor histidine kinase/response regulator Fos-1/TcsA
AFLA_026290	*ypd1*	113.21	142.22	0.32	phosphotransmitter protein Ypd1, putative
AFLA_099770		27.69	22.64	−0.29	conserved hypothetical protein
AFLA_116790		3.46	3.40	−0.02	conserved hypothetical protein
AFLA_126140		0.27	0.30	0.15	conserved hypothetical protein
AFLA_050640		25.73	14.39	−0.83	dual specificity phosphatase catalytic domain protein
AFLA_134840	*tep1*	20.29	9.44	−1.10	phosphoinositide phosphatase Pten/Tep1, putative
AFLA_133310		34.00	30.07	−0.17	protein-tyrosine phosphatase, putative
AFLA_087770	*spc105*	16.23	14.73	−0.14	chromosome segregation protein Spc105, putative
AFLA_043340	*spc19*	43.75	42.86	−0.02	mitotic spindle biogenesis protein Spc19, putative
AFLA_086270	*msn5*	11.87	16.02	0.43	nuclear import and export protein Msn5, putative
AFLA_053070		27.66	33.94	0.29	Yapsin, putative
AFLA_021190		26.06	24.19	−0.10	PAP2 domain protein
AFLA_056030		38.60	36.91	−0.06	PAP2 domain protein
AFLA_084240		89.81	92.17	0.03	poly(A) polymerase Pap
AFLA_095910		27.53	31.57	0.19	PAP2 domain protein
AFLA_135870		37.63	54.54	0.53	PAPA-1-like conserved region protein
AFLA_048050	*nap1*	408.00	403.31	−0.01	nucleosome assembly protein Nap1, putative
AFLA_092040		23.44	25.05	0.09	NAP1-binding protein, putative

* CK=Control; D1=1% dimethylformamide.
